# A Two-Phase Time Synchronization-Free Localization Algorithm for Underwater Sensor Networks

**DOI:** 10.3390/s17040726

**Published:** 2017-03-30

**Authors:** Junhai Luo, Liying Fan

**Affiliations:** School of Electronic Engineering, University of Electronic Science and Technology of China, Chengdu 611731, China; liy_fan@std.uestc.edu.cn

**Keywords:** underwater sensor networks, synchronization-free, range-free, particle swarm optimization

## Abstract

Underwater Sensor Networks (UWSNs) can enable a broad range of applications such as resource monitoring, disaster prevention, and navigation-assistance. Sensor nodes location in UWSNs is an especially relevant topic. Global Positioning System (GPS) information is not suitable for use in UWSNs because of the underwater propagation problems. Hence, some localization algorithms based on the precise time synchronization between sensor nodes that have been proposed for UWSNs are not feasible. In this paper, we propose a localization algorithm called Two-Phase Time Synchronization-Free Localization Algorithm (TP-TSFLA). TP-TSFLA contains two phases, namely, range-based estimation phase and range-free evaluation phase. In the first phase, we address a time synchronization-free localization scheme based on the Particle Swarm Optimization (PSO) algorithm to obtain the coordinates of the unknown sensor nodes. In the second phase, we propose a Circle-based Range-Free Localization Algorithm (CRFLA) to locate the unlocalized sensor nodes which cannot obtain the location information through the first phase. In the second phase, sensor nodes which are localized in the first phase act as the new anchor nodes to help realize localization. Hence, in this algorithm, we use a small number of mobile beacons to help obtain the location information without any other anchor nodes. Besides, to improve the precision of the range-free method, an extension of CRFLA achieved by designing a coordinate adjustment scheme is updated. The simulation results show that TP-TSFLA can achieve a relative high localization ratio without time synchronization.

## 1. Introduction

Underwater Sensor Networks (UWSNs) are usually composed of some autonomous and individual sensor nodes [1], which can sense data, perform intelligent computations, and forward information. Sensor nodes are spatially distributed in UWSNs with some sensing work to obtain water-related properties such as mass, temperature and pressure data [2]. UWSNs usually arrange many sensor nodes to monitor the underwater environment. Those sensor nodes exchange the node location information and other data through the underwater acoustic communication. Moreover, UWSNs can be applied to many areas such as disaster early warning, pollutant control, marine resource exploration and maritime military activity.

The location information of the sensor nodes in UWSNs is necessary to provide users with an efficient testing service. Therefore, underwater sensor node positioning can be regarded as the foundation and core for UWSNs. How to accurately estimate the position of the underwater node in UWSNs is of great research significance. Many researchers have studied the topic of sensor localization. In an outdoor environment, GPS-based positioning systems are mostly used and have a good performance. In an indoor environment, RF-based and VLC-based positioning systems have attracted many researchers. However, it is unfeasible to apply any of them to UWSNs. Only RF signals at low frequencies of about 30–300 Hz can be used in UWSNs, moreover, large antennas or high transmission power is needed [3]. Optical signals are also subject to underwater attenuation and scattering [4]. Fortunately, the frequency of sound waves is small between 10 Hz and 1 MHz [1], which can provide small bandwidth but long wavelengths. Therefore acoustics can be used to relay information over kilometers [5].

Underwater localization usually requires some objects with known locations (anchors) and objects to be localized (unknown nodes) [6]. The location information of anchors can be obtained through a variety of methods. In [7], the authors divided the localization scheme of UWSNs into two phases, namely the position-related information collection phase and the position estimation phase. In the first phase, position-related information such as the distance, angle, and hop count between each other or the anchor point is measured by the node. In the second phase, the localization algorithms are performed by the sink nodes or locally calculated by them. Conventional localization algorithms use the distance or angle measurements between the anchor and the unknown nodes to estimate the location of unknown nodes. Some positioning schemes do not require an anchor node and use the connection information to obtain the location of unknown nodes [8,9]. The deployment of UWSNs is still a challenging task because of the limitations of computing power, cost, memory, transmission range and the lifetime of any single sensor [1]. A large number of anchor nodes can provide greater coverage and higher accuracy but may add cost. Therefore, how to decrease the number of anchor nodes or to achieve anchor-free localization is still an active research direction. Moreover, the battery resources are limited which shortens the operation time. Thus, an effective strategy must guarantee the system performance with low energy consumption.

Time synchronization is directly assumed in many localization schemes. However, it is not feasible in the UWSNs. In [10], the authors show that precise time synchronization is hard to achieve due to the characteristics of sound. In UWSNs, the propagation delay is much higher due to the five-orders-of-magnitude difference in the speed of sound in water compared with RF propagation: 1500 m/s compared with $3\times 10^{8}$ m/s. Therefore the high propagation delay of UWSNs will bring time synchronization problems. Many time synchronization localization algorithms rely on some form of clock synchronization, either by ensuring that the transmitter-receiver is synchronized with the two-way message or by assuming that surface “anchor” nodes which can synchronize their clocks and disseminate or collect information to or from other nodes [11]. However, that method will bring new challenges in terms of overhead and communications management, and often assumes very low data rate communications. Therefore how to develop a synchronization-free algorithm is a direction to solve this problem.

In this paper, basing on Time Synchronization-Free Localization using mobile beacons (we called it as TSFL) [12], we propose the Two-Phase Time Synchronization-Free Algorithm (TP-TSFLA). TP-TSFLA can be divided into two phases, namely, a range-based estimation phase (Phase I) and range-free evaluation phase (Phase II). In Phase I, we use the TSFL algorithm to obtain the distance measurements from the anchor node to the unknown sensor nodes, then we employ the PSO algorithm to solve the localization optimization problem. In Phase II, we propose a range-free algorithm to locate the unlocalized sensor nodes. Only sensor nodes which cannot be localized in Phase I will execute Phase II. In Phase II, the localized sensor nodes act as the new anchor nodes to help realize localization. The unlocalized sensor node actively initiates a localization request, then the localized sensor nodes within the transmission range of the unlocalized sensor node can receive the request and respond their coordinate to the unlocalized node. Then the unlocalized sensor node starts a Circle-based Range-Free Localization Algorithm (CRFLA) to locate itself. Besides, a coordinate adjustment scheme is proposed to improve the precision of CRFLA. In our algorithm, we use the TSFL algorithm to obtain the distance measurements without time synchronization. In the TSFL algorithm, the distance is estimated by using the time interval between the first and second message received by the unknown sensor node from the same anchor node. Thus, the time synchronization between the sensor node and anchor node is unnecessary. Also, the synchronization among the unknown sensor nodes or the anchor nodes is unnecessary. It is obvious that the PSO algorithm and CRFLA do not have the requirement of time synchronization. Therefore, TP-TSFLA we proposed in this paper without time synchronization can effectively solve the problem of time synchronization.

The remaining portion of this paper is organized as follows: in Section 2, we survey localization algorithms according to their different natures. The system model is given in Section 3. In Section 4, we use the range-based estimation algorithm based on the PSO algorithm to obtain the coordinates of the unknown sensor nodes. CRFLA and its coordinate adjustment scheme are presented in Section 5 and Section 6, respectively. The detailed algorithm application procedure is shown in Section 7. Section 8 shows the simulation results and comparison. Finally, we conclude in Section 9.

## 2. Related Work

Recently, numerous localization algorithms have been put forward, and some researchers have done some surveys of localization algorithms [13,14]. In this section, we discuss the localization algorithms in UWSNs in five aspects, namely computation algorithms, anchor requirements, range measurements, synchronization requirements, and communication between nodes. We only discuss the differences in each aspect and use some references to describe them.

According to the computation algorithm to be implemented, we classify the computation algorithms into two categories: centralized techniques [15] and distributed techniques [16]. The centralized techniques perform the localization algorithm at the command center or the sink node. However, in the distributed techniques, the sensor nodes alone estimate the location of each sensor node. In [17], a Reverse Localization Scheme (RLS) with a fast response to events is proposed. The scheme is based on the centralized technique. Thus, the data can be transmitted to the station, and the positioning algorithm is executed there. The scheme is divided into two phases, namely, a a transmitting phase and a centralized geometric localization phase. In the transmission phase, a new message exchange mechanism based on event-driven reporting is proposed. At the beginning of the second phase, the sink collects information from the anchor and estimates the location of the sensor node. The authors of [18] have reported a localization algorithm based on distributed technology. The authors mainly consider the problem of estimating the isolated unknown nodes and propose a Multihop Fitting Localization Approach (MFLA). The method sets the intermediate node between the beacon and the unknown node as a router to construct the path through the greedy method, and then fits the multi-hop path into a straight line and estimates it by trilateration.

The anchor requirement means the anchor node is required or not in the localization algorithm. According to this, we classify the localization algorithms into two taxonomies: anchor-free and anchor-based schemes. In UWSNs, many positioning algorithms can use anchor nodes to help estimate location. However anchor nodes are not necessary, and some researchers have proposed a self-localization algorithm that does not need anchor nodes. The positioning scheme [19] is an anchor-based scheme. This scheme consists of four types of nodes: surface buoys, Detachable Elevator Transceivers (DETs), anchor nodes and ordinary nodes. Besides, the scheme locates the nodes in two phases. First, the anchor node uses a range-based distributed approach to locate itself. Secondly, ordinary nodes use the regional positioning scheme to achieve location-free centralized approach. In [20], an Anchor-Free Location Algorithm (AFLA) for active restricted UWSNs is proposed. The algorithm uses the relationship between adjacent nodes. In this scenario, the underwater sensor node which is actively limited means that when anchored to the seafloor, it floats in the sea and moves within the hemispherical region. A node with unknown location broadcasts a message and receives the information from other nodes at the same time. When the node receives two messages from two different nodes, it starts the location calculation process.

Based on the range measurement, we classify the localization algorithms into two categories: range-based schemes, and range-free schemes. In general, range-based schemes estimate distances by various algorithms and then convert them into positional information. A range-free scheme does not require distance measurementa and bearing information, but uses a local topology and the position of the neighboring anchor nodes to obtain the position estimate. However, the range-free scheme can only get a rough location with little accuracy. The positioning method of [20] is a range-based approach and is known as the multi-stage AUV-assisted positioning scheme that is an improvement of the “multi-stage DNR” scheme. In [16], the DNR is replaced by an AUV. The AUV with known coordinates dives to a pre-programmed depth and begins to traverse the sensor network according to a pre-programmed path. When a non-collinear position receives signals from three beacons, triangulation is used to obtain the position of the node. In [21], the authors propose an efficient Area Localization Scheme (ALS). The scheme estimates the position of the sensor within a particular region. An anchor node broadcasts a beacon signal to a sensor node and sends an acoustic signal with a varying power level. The sensor nodes passively monitor the signals and record the received information, then forward it to the sink. The sink uses the information gathered from sensor nodes to estimate the area in which the sensor is localized.

The synchronization requirement means that time synchronization is required or not in the localization algorithm. Based on this, we classify the localization algorithms into two categories: synchronization localization schemes and the synchronization-free localization schemes. In many cases, the localization scheme directly assumes that sensor nodes are synchronized with each other. However, this is difficult to implement in underwater environments, thus the researchers have proposed some localization algorithms without synchronization requirements. The localization scheme of [22] requires time synchronization, but dual hydrophones on each node can reduce the time synchronization requirement. In [22], a Dual Hydrophone Localization (DHL) approach is proposed, and the localization problem is converted into a half-plane intersection problem. As for the synchronization-free positioning scheme, we introduce three papers to show it. In [23,24], a range-free scheme using AUV periodically broadcasts message blocks via four directional beams to estimate the location information of sensor nodes. The node receives the message block and uses two different continuous beams to estimate the position of the AUV at two different moments. The location of the nodes can then be obtained by using the two estimated locations. In [25], a Basic Synchronization-Free Localization (BSFL) scheme is proposed. It consists of two steps, namely the range difference calculation, and the position calculation. However, the BSFL still suffers from some of the drawbacks of large-scale UWSNs. Therefore, a Large-Scale Localization Scheme (LSLS) based on BSFL is designed. It consists of three phases, namely sea surface anchoring, iterative localization and complementary phase.

Based on the communication characteristics between the reference node and the common node, we classify the UWSNs localization algorithm into two classes: single stage methods [26,27] and multi-stage methods [28,29,30]. The single stage method means that the exchange of messages between all sensor nodes and the reference nodes is straightforward. After obtaining the location, they are still passive and cannot be used to help locate other sensor nodes [31]. In the multi-stage scheme, the common nodes do not need to communicate directly with the reference nodes. Once sensor nodes are localized, they become new reference nodes and can help to locate other sensor nodes [31]. The positioning scheme of [32] is based on the single-stage method. The authors use hyperbolic methods and normal distribution estimation error modeling and calibration for location estimation. The positioning scheme [33] is based on a multi-level localization scheme. The Top-down Positioning Scheme (TPS) for UWSNs without evenly distributed anchor nodes or additional infrastructures can increase location coverage while maintaining low positioning errors. In this scheme, there are three types of nodes, namely surface anchor nodes, new reference nodes, and non-localized nodes. First, only sensor nodes that are close to the surface anchor nodes can be localized. Once the positions of sensor nodes are obtained, they compute their confidence values and compare them to the confidence thresholds. If the nodes’ confidence values are greater than the confidence thresholds, they become new reference nodes to help the non-localization nodes locate themselves.

Next, we compare the TP-TSFLA we proposed in this paper (we use the term “our” to express it in Table 1) and those algorithms which are mentioned in Section 2 in the five aspects (computation algorithm, anchor requirement, range measurement, synchronization requirement, and communication between nodes). The detailed comparison is shown in Table 1.

## 3. System Model

### 3.1. Overview of the System

This paper mainly concentrates on locating the underwater sensor nodes. Due to the unfeasibility of the assumption of perfect time synchronization, we propose TP-TSFLA to realize localization without time synchronization. In this algorithm, sensor nodes are randomly deployed in the different depth of the underwater to monitor various areas. We assume sensor nodes are static within the 3D-network architecture. A pressure sensor is equipped on every sensor node to obtain the depth of the sensor node as the z-coordinate. Obtaining the x-coordinate and y-coordinate of the sensor node is necessary. Hence, the 3D-localization problem can be transformed into a 2D-localization issue.

**Figure 1:**
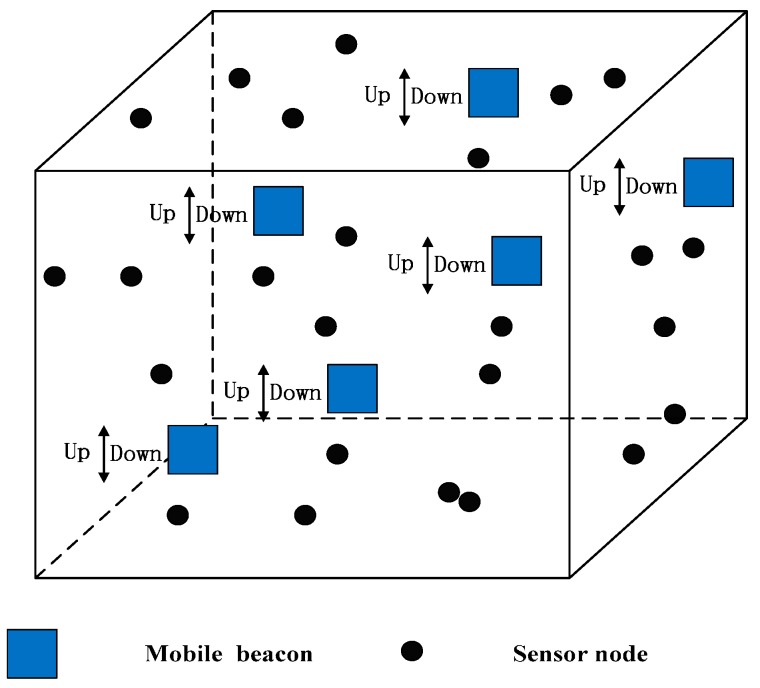
System model.

To obtain the coordinates of the sensor node, some particular nodes which the coordinate that can be looked as known are needed. In this scheme, we use a mobile beacon to help realize localization, and no other anchor nodes are required. A mobile beacon can dive and rise in the vertical direction with the aid of extra weight. When it reaches the deepest point of the deployment, it rises to the surface. Once it floats on the sea surface, it can use a GPS receiver to obtain its new coordinates. Hence, when the mobile beacon dives into the deepest deployment point, we suppose that only the the z-coordinate is changed over time. Also, the mobile beacon can use a pressure sensor to obtain the z-coordinate. The detailed deployment scheme is shown in Figure 1.

All the mobile beacons have a fixed transmission range and a fixed diving speed and can broadcast messages at fixed time intervals. The mobile beacons broadcast messages which contain the mobile beacon id and coordinates. Sensor nodes in the transmission range of the mobile beacon can receive the message. Then sensor nodes can use the geometric properties to locate themselves. During this phase, the sensor node only passively listens to the message from the mobile beacon to decrease the power consumption. After that, the unlocalized sensor node actively launches the localization request. The localized sensor node which is in the transmission range of the unlocalized sensor node acts as the new anchor sensor node and responds with the coordinates to the unlocalized sensor node. The unlocalized sensor node uses CRFLA to locate itself.

### 3.2. Time Synchronization-Free Localization Scheme Using Mobile Beacons

Based on the system model, we can employ the time synchronization-free localization scheme proposed in [12] to obtain the distance measures in Phase I. Hence, to describe it concisely, we called it as the TSFL algorithm. The mobile beacon dives and rises in the underwater at the fixed speed $v_{1}$. $T_{1}$ and $T_{2}$ express the time that the first message received by sensor node and the second message received by the sensor node respectively. Hence, the coordinates of the mobile beacon at the different times are denoted as $\left(x_{1},y_{1},z_{1}\right)$ and $\left(x_{1},y_{1},z_{2}\right)$. The speed of sound is $v_{2}$, and the coordinate of the sensor node is $\left(x,y,z_{3}\right)$. The distance measure from the mobile beacon to the sensor is expressed as d.

If $z_{1}<z_{3}<z_{2}$, we can obtain the distance d, and the detailed process can be found in [12]:

(1) $$d=\sqrt{\frac{1}{4}{\left(\left(\frac{A_{1}}{B_{1}}\right)+B_{1}\right)}^{2}-H_{1}^{2}}$$

where $H=z_{2}-z_{1}$, $H_{1}=z_{3}-z_{1}$, $H_{2}=z_{2}-z_{3}$, $A_{1}=H_{1}^{2}-H_{2}^{2}$, and $\Delta T=T_{2}-T_{1}$, $B_{1}=v_{2}\left(\frac{H}{v_{1}}-\Delta T\right)$.

If $z_{1}<z_{2}<z_{3}$, we can obtain the distance d by using the following equation:

(2) $$d=\sqrt{\frac{1}{4}{\left(\left(\frac{A_{2}}{B_{2}}\right)+B_{2}\right)}^{2}-H^{2}}$$

where $H=z_{3}-z_{1}$, $H_{1}=z_{2}-z_{1}$, $H_{2}=z_{3}-z_{2}$, $A_{2}=H^{2}-H_{2}^{2}$, and $B_{2}=v_{2}\left(\frac{H_{1}}{v_{1}}-\Delta T\right)$, $\Delta T=T_{2}-T_{1}$.

If $z_{3}<z_{1}<z_{2}$, we can obtain the distance d by using the following equation:

(3) $$d=\sqrt{\frac{1}{4}{\left(\left(\frac{A_{3}}{B_{3}}\right)+B_{3}\right)}^{2}-H_{1}^{2}}$$

where $H=z_{2}-z_{3}$, $H_{1}=z_{1}-z_{3}$, $H_{2}=z_{2}-z_{1}$, $A_{2}=H_{1}^{2}-H^{2}$ and $B_{2}=v_{2}\left(\frac{H_{2}}{v_{1}}-\Delta T\right)$, $\Delta T=T_{2}-T_{1}$.

If at least three distance measures from different mobile beacons have been obtained, the position of the sensor node can be obtained. However, the authors do not take the impact on the water current into account and make the speed of the mobile beacon and sound a constant. This is not actually held true. Thus, we consider the error caused by the underwater environment and propose a localization scheme based on the PSO algorithm. Besides, to save costs, the number of mobile beacons is limited, which leads to a lower localization ratio. Especially when we use the algorithm in the relatively large environment, the localization ratio is not enough. Hence, we improve the algorithm based on the two aspects in TP-TSFLA.

### 3.3. Algorithm Features

In this paper, TP-TSFLA is mainly concerned with the time synchronization requirements, trying to find a synchronization-free localization scheme. The localization algorithm proposed in this paper is based on the distributed localization technique. In Phase I, mobile beacons are used as anchor nodes, and in Phase II, sensor nodes that are localized in the first phase act as anchors to help locate the unlocalized sensor nodes. The algorithm used in Phase I is range-based, while the algorithm of Phase II is range-free, and belongs to the multi-stage method. The features of the system can be described as follows.

- The system is suitable for using in a 3D-network architecture, and the sensor node is assumed to be static in the network. Every sensor node is equipped with a pressure sensor to sense its depth. The mobile beacons can obtain their x-coordinate and y-coordinate by GPS, and only the z-coordinate is changed when the mobile beacon dives in the sea.
- The diving speed of the mobile beacon and the rate of the sound in the water of the TSFL algorithm are assumed as a constant. The mobile beacon broadcasts the message at a fixed interval. The transmission range of the mobile beacon and sensor node is fixed. The transmission range of the mobile beacon is larger than the transmission range of sensor nodes.
- During Phase I, sensor nodes passively listen to the mobile beacon without transmitting a message to the mobile beacon to decrease the power consumption, while in Phase II, sensor nodes can initiate active communication with other sensor nodes to obtain the message which is required to realize localization.
- In TP-TSFLA, the mobile beacons are used as the anchor nodes. In Phase II, the algorithm uses a multi-stage scheme to help realize the localization. The localized sensor nodes are used as the new anchor nodes.

## 4. Range-Based Estimation Algorithm Using PSO

In this section, we employ the Particle Swarm Optimization (PSO) algorithm to obtain the estimated position of the unknown sensor nodes. To solve a variety of optimization problems, many optimization algorithms have been proposed, such as climbing method, genetic algorithm and so on. The hill climbing method has high precision, but it easily falls into a local minimum. The genetic algorithm belongs to the evolutionary algorithm class. However, the genetic algorithm requires more sophisticated programming, and the choice of the parameters severely affects the quality of the solution, and most of these parameters depend on experience. The PSO algorithm, with smooth implementation, high precision, and fast convergence is similar to a genetic algorithm, and it also starts from a random solution. The PSO algorithm iteratively finds the optimal solution and evaluates the quality of the solution through fitness, but it is simpler than the genetic algorithm. It does not have the “cross” (crossover) and “mutation”. The global optimum is sought by following the current search to the optimal value.

Basing on the TSFL algorithm, after at least three distance measures $d_{i}$ from different mobile beacons $\left(x_{i},y_{i},z_{i}\right)$ have been obtained, the authors of [12] estimate the position of the sensor node (x,y,z) by using the following equation:

(4) $$X=\left[\begin{array}{l} x\\ y \end{array}\right]={\left(A^{T}A\right)}^{-1}A^{T}b$$

where:

(5) $$A=\left[\begin{array}{c} 2(x_{n}-x_{1})\;2(y_{n}-y_{1})\\ 2(x_{n}-x_{2})\;2(y_{n}-y_{2})\\ \vdots \\ \vdots \\ 2(x_{n}-x_{n-1})\;2(y_{n}-y_{n-1}) \end{array}\right]$$

and:

(6) $$b=\left[\begin{array}{c} d_{1}^{2}-d_{n}^{2}-x_{1}^{2}-y_{1}^{2}+x_{n}^{2}-y_{n}^{2}\\ d_{2}^{2}-d_{n}^{2}-x_{2}^{2}-y_{2}^{2}+x_{n}^{2}-y_{n}^{2}\\ \vdots \\ \vdots \\ d_{n-1}^{2}-d_{n}^{2}-x_{n-1}^{2}-y_{n-1}^{2}+x_{n}^{2}-y_{n}^{2} \end{array}\right]$$

However, this kind of method to obtain the coordinate of the unknown sensor node is not suitable for our algorithm. When we take the impact on changing speed for the mobile beacon and for the sound through the water, the localization problem becomes a multidimensional non-linear optimization problem, and we employ the PSO algorithm to solve it. The detailed mathematical derivation is shown as follows.

The distance between unknown sensor node (x,y,z) and the anchor node $\left(x_{i},y_{i},z_{i}\right)$ expressed as $r_{i}$ is given as:

(7) $$\begin{array}{l} \left\{ \begin{array}{c} \sqrt{{\left(x_{1}-x\right)}^{2}+{\left(y_{1}-y\right)}^{2}+{\left(z_{1}-z\right)}^{2}}=r_{1}\\ \sqrt{{\left(x_{2}-x\right)}^{2}+{\left(y_{2}-y\right)}^{2}+{\left(z_{2}-z\right)}^{2}}=r_{2}\\ \vdots \\ \sqrt{{\left(x_{n}-x\right)}^{2}+{\left(y_{n}-y\right)}^{2}+{\left(z_{n}-z\right)}^{2}}=r_{n} \end{array}\right. \end{array}$$

Let ${\hat{r}}_{i}$ be the noisy range value of $r_{i}$. Actually, in this paper, the value of ${\hat{r}}_{i}$ is the distance measure $d_{i}$ which is obtained by using the TSFL algorithm. Then:

(8) $$d_{i}={\hat{r}}_{i}=r_{i}+n_{i}$$

where $n_{i}$ is the measured noise.

Assume that the estimated location of the unknown sensor nodes is x, and the location of the anchor node is p, then the estimated location of the unknown sensor nodes x is given by:

(9) $$x=\arg\underset{x}{\min}\sum_{i=1}^{N}\left(d_{i}-||p_{i}-x||\right)$$

where $\left||p_{i}-x|\right|$ represents the Euclidean distance between $p_{i}$ and x. N is the number of anchor nodes which can be received by the sensor node. Thus, the localization is a multidimensional non-linear optimization problem which can only be solved by using an iterative approach [34]. Equation (9) involves a non-convex objective function (with a Hessian matrix not positive definite). Therefore, the result of using the L2 optimization methods is not generally optimal. To solve this problem with low computational cost, we employ the PSO algorithm. Besides, the PSO algorithm can ensure high accuracy.

### 4.1. Search Space of Particles

The transmission range of anchor node $\left(x_{i},y_{i},z_{i}\right)$ is *R*. In [35], the authors use the circumscribed square of side length R as the search space of particles. Then:

(10) $$S_{i}=[x_{i}-R,x_{i}+R]\times [y_{i}-R,y_{i}+R]$$

where $S_{i}$ is the search space of the particles. If the unknown sensor node has N anchor nodes, the search space of the particles can be expressed as:

(11) $$S=\overset{N}{\underset{i=1}{\cap }}S_{i},$$

Actually, the transmission range of an anchor node is a circle of the radius *R*, thus we redesign the search space of particles. The search space of an anchor node $S_{i}$ is a circle of the radius *R* and circle center $\left(x_{i},y_{i}\right)$, moreover, $S_{i}$ can be expressed as follows:

(12) $${\left(x-x_{i}\right)}^{2}+{\left(y-y_{i}\right)}^{2}=R^{2}$$

In our algorithm, at least three anchor nodes are needed to obtain the coordinate of the unknown sensor node. Therefore, the search space of the particles S is given as:

(13) $$S=\overset{N}{\underset{i=1}{\cap }}S_{i}$$

Hence, we initialize a group of random particles (random candidate solution) in the search space of the particles S. Then the PSO algorithm iteratively finds the optimal solution. In each iteration, the particle updates itself by tracking two extreme values. One of the extreme values is the optimal solution found by the particle itself, and the solution is called the individual extreme (pBest). The other is the optimal solution found by the whole population. The extreme value is the global extreme value (gBest). When these two optimal values are found, the particle updates its speed and new position according to the following equation:

(14) $$v[]=w\times v[]+c_{1}\times rand()\times (pbest[]-present[])+c_{2}\times rand()\times (gbest[]-present[])$$

(15) prsent[]=present[]+v[]

where v[] is the speed of the particle, w is the inertia weight, present[] is the current position of the particle, pbest[] is the individual extreme value, gbest[] is the global extreme value, and rand() is the random number between (0, 1). $c_{1}$ and $c_{2}$ are the learning factors.

### 4.2. Inertia Weight 

In general, the inertia weight can be fixed or linear decrease. Moreover, some studies have shown that bigger inertia weight contributes global search, while smaller inertia weight contributes local search [36]. In [36], the authors use the following equation to express the inertia weight:

(16) $$\omega =\omega_{\max}-\frac{\omega_{\max}-\omega_{\min}}{k_{\max}}\times k,$$

where, $\omega_{\max}$ is the initial weight, $\omega_{\min}$ is the final weight, $k_{\max}$ is the maximum number of iterations, and k is the current iteration. In this paper, we use the same parameters as [36].

### 4.3. Learning Factor

The learning factor can be a constant or variable. In [36], the authors give formulas for $c_{1}$ and $c_{2}$, respectively:

(17) $$c_{1}=1.3+1.2\times \cos(t),t\in [0,\pi ]$$

(18) $$c_{2}=2.0-1.2\times \cos(t),t\in [0,\pi ]$$

(19) $$t=\frac{\pi }{k_{\max}}\times k$$

where $k_{\max}$ is the maximum number of iterations, and k is the current iteration.

The design of the learning factor based on that global search does not easily fall into local optimum at the beginning of the search. Thus, in earlier search, $c_{1}$ with a larger value, and $c_{2}$ with a smaller value will enhance the global search capability of the PSO algorithm. In the latter part of the search, $c_{1}$ with a smaller value and $c_{2}$ with a larger value will enhance the local search capabilities of the PSO algorithm.

### 4.4. Fitness Function 

The distance measure of the unknown sensor node (x,y,z) from the anchor node $\left(x_{i},y_{i},z_{i}\right)$ is $d_{i}$. The coordinate of the particle is $\left(x_{j},y_{j},z_{j}\right)$, and the number of anchor node is N, and the fitness function can be described as:

(20) $$f_{j}=\sum_{i=1}^{N}\left|d_{i}-\sqrt{{\left(x_{j}-x_{i}\right)}^{2}+{\left(y_{j}-y_{i}\right)}^{2}+{\left(z_{j}-z_{i}\right)}^{2}}\right|,$$

If the fitness function tends to be 0, the estimated coordinate tends to be the coordinate of the unlocalized sensor node. After the maximum number of loops is reached, the current global extreme value will be chosen as the coordinate of the sensor node.

## 5. CRFLA

**Figure 2:**
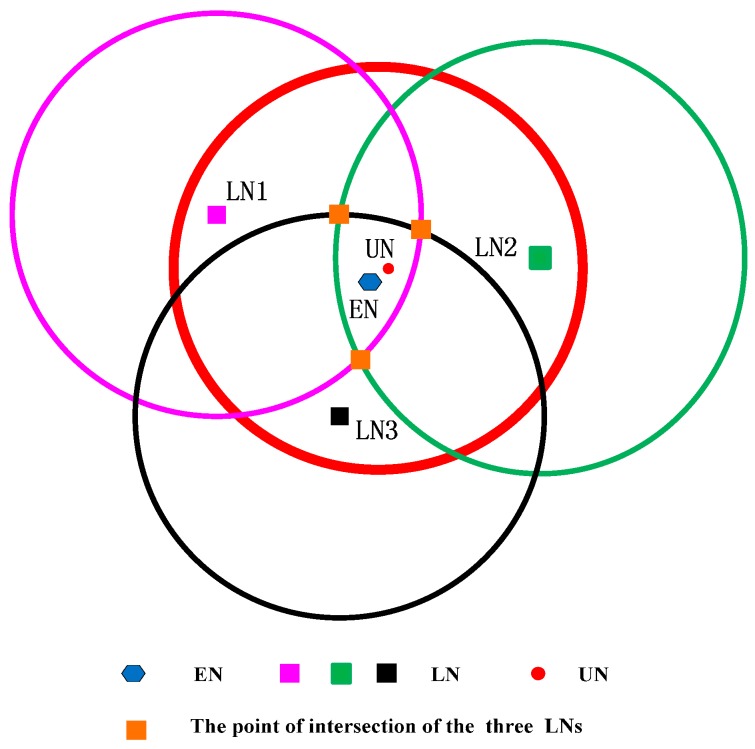
CRFLA.

Generally, the number of the anchor nodes is limited when we take the cost of the system into account. Actually, in our simulation environment, the number of mobile beacons is 25, while the number of unknown sensor nodes is 800. Although the anchor nodes have a large transmission range, there still are some unknown sensor nodes which cannot receive at least three pieces of information from different anchor nodes. Therefore, after Phase I, some sensor nodes may not obtain the location information. Then those sensor nodes execute Phase II to locate themselves. In Phase II, the environment is different from Phase I. In Phase I, the mobile beacon acts as the anchor node, and the number of mobile beacons is small. However, in Phase II, the localized node serves as the new anchor node, and the number is much more than the unlocalized sensor node. The unlocalized sensor node (UN) transmits the localization request, and the localized sensor node (LN) which is localized in the transmission range of UN (denoted as r) can receive the request. The distance $d_{p}$ between UN and LN which is in the transmission range satisfies the inequation $d_{p}<r$. We draw a circle whose center is the localized sensor node $\left(x_{p},y_{p},z_{p}\right)$ and which radius is r, then the unlocalized sensor node must locate in the circular area. In the range-base algorithm, we can use the point of intersection of those three circles as the estimated coordinate of the sensor node (EN), but in the range-free algorithm, the three circles may not intersect at one point. Thus, we use the geometric center of the intersection area of the circles as the coordinate of the unlocalized sensor node (shown in Figure 2). We can see that if the intersection area is small, the precision will be much higher. We can state that if the following two conditions are satisfied, the precision of the circle-based range-free algorithm will be much higher.

*Condition I*: The distance $d_{p}$ between the unlocalized sensor node and the localized sensor node is infinite close to the transmission range r of the unlocalized sensor node.

*Condition II*: The three localized sensor nodes locate in the different directions of the circle.

**Figure 3:**
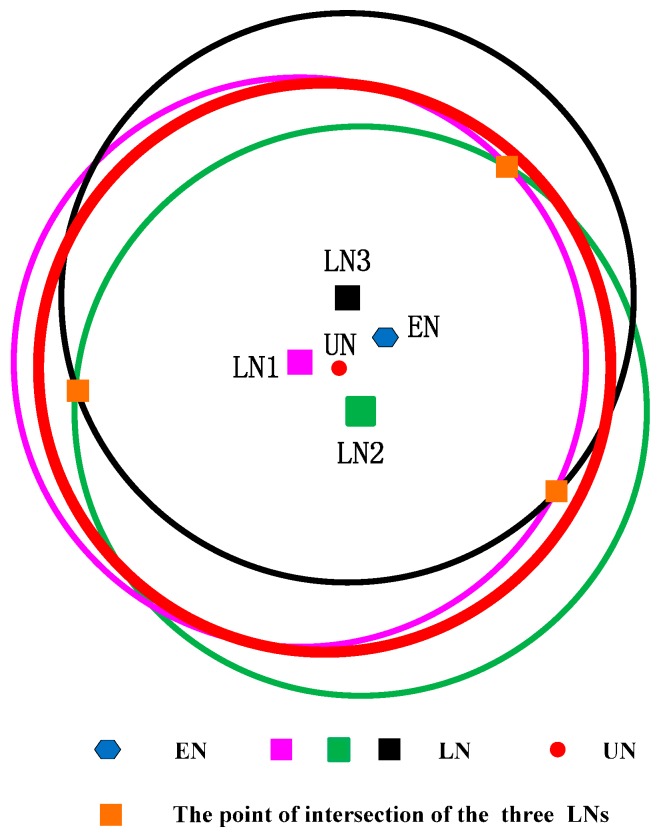
CRFLA against Condition I.

Here, we use the figure to show the counter-example of the two conditions. We assume $d_{p}<r/2$ to verify Condition I and show it in Figure 3.

As shown in Figure 3, if $d_{p}$ is much smaller than r, the intersection area will increase. If the three intersection points are symmetrical, the geometric center of the intersection area is still near the unlocalized sensor node. However, all sensor nodes are randomly distributed in UWSNs, and the probability of the three localized sensor nodes being distributed symmetrically is very low. Hence, if the intersection area is small, even though the three localized sensor nodes are not symmetrical, the geometric center will not be far away from the unlocalized sensor node.

To satisfy Condition I as far as possible, we employ the fact that the signal strength decreases with the increase of distance. The unlocalized sensor node utilizes the response information from the localized sensor node which contains the coordinates of the localized sensor node and the signal strength to choose the three localized sensor nodes. Simply said, the unlocalized sensor node determines the three localized nodes which have the lowest signal strength. It means that the distance of the chosen three localized sensor nodes from the unlocalized sensor node is the largest in all localized sensor nodes which can receive the information from the unlocalized sensor node. Here, we do not obtain the distance from the signal strength but compare the value of the signal strength.

**Figure 4:**
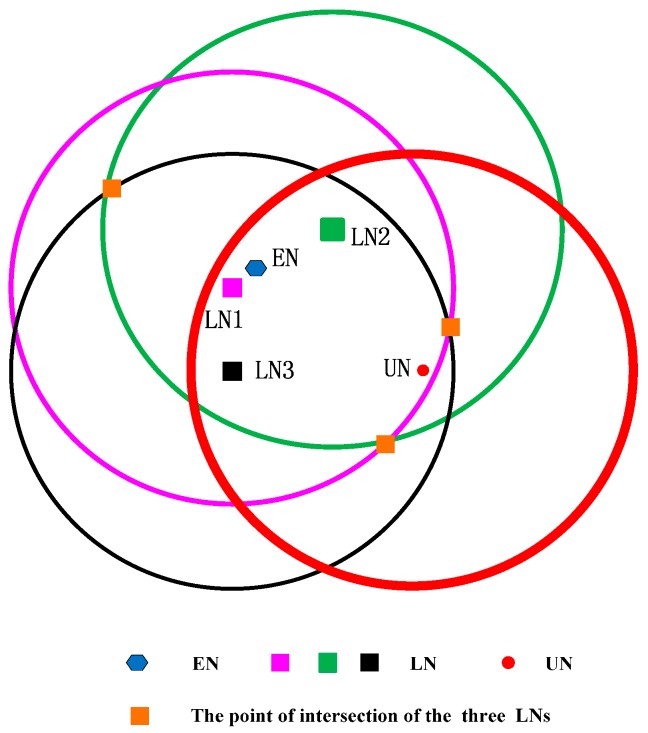
CRFLA against Condition II.

We suppose the following case that the three localized sensors satisfy Condition I but not satisfy Condition II, and show it in Figure 4.

Three localized sensor nodes are far away from the unlocalized sensor node and $d_{p}$ is close to r, but the three localized sensor nodes are in the same direction of the circle of the unlocalized sensor node. In Figure 4, the intersection point is three, and the geometric center of the intersection area is far away from the unlocalized sensor node. It means that the localization error is larger.

To satisfy Condition II as far as possible, we employ the k-means clustering algorithm. The clustering algorithm can ensure that the class distance is as small as possible, and the distance between classes as large as possible. In TP-TSFLA, we cluster the localized node which locates in the transmission range of the unlocalized sensor node into four categories. The localized sensor node with the lowest signal strength in each category is chosen. It means that four localized sensor nodes with the lowest signal strength in four different categories are determined. Any three of the four localized sensor nodes are picked. We use the localized sensor node as the circle center and r as the radius to draw the circle. Hence, three circles can be obtained, and then calculate the geometric center of the intersection area of the three circles. Using the four localized sensor nodes, we can get four different groups of intersection area.

**Figure 5:**
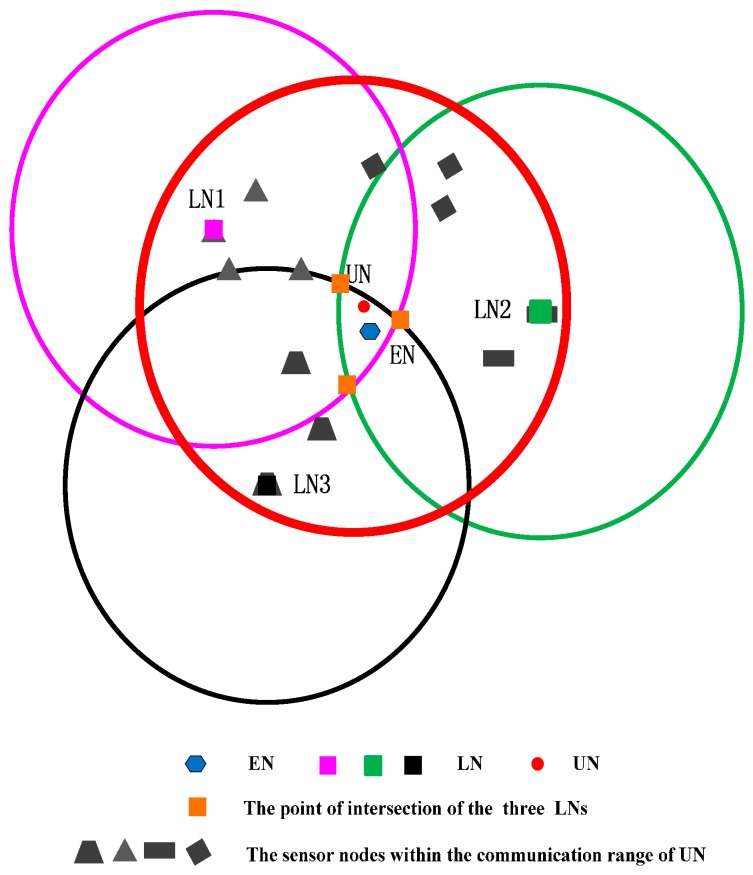
The detailed procedure of CRFLA.

Then the average value of the four geometric centers of the four intersection areas is used as the coordinate of the unlocalized sensor node. The circle-based range-free algorithm is shown in Figure 5.

The red circle expresses the transmission range of the unlocalized sensor node. The other points within the red circle mean the localized sensor node which can receive the request of the unlocalized sensor node. Then employing the k-means algorithm clusters those localized sensor nodes into four classes (using different shapes to express). In each class, the algorithm picks out the LN which is the lowest signal strength in its cluster (the distance between the localized sensor nodes and unlocalized sensor nodes is the largest). Three LNs draw three circles. The geometric center of the intersection area of the three circles can be obtained.

If LNs within the transmission range of UN satisfy the two conditions with a high possibility to ensure that the three circles are intersecting and the intersection area is relatively small. The relatively high precision can be guaranteed. Note that the intersection point can be three or two. We can describe CRFLA in Algorithm 1.
**Algorithm 1:** CRFLA**Step 1:** The unlocalized sensor node (UN) (x,y,z) transmits the localization request.**Step 2:** The localized sensor nodes (LNs) which satisfy $d_{p}<r$ respond the information which contains the coordinate $\left(x_{p},y_{p},z_{p}\right)$ and signal strength RSSIp to the UN.**Step 3:** The UN use the k-means clustering algorithm to cluster the LNs into four classes ($cc_{i}$).**Step 4:** For each cci, choosing the LN with $\min(RSSI_{p})$, four LNs can be obtained as LN1,LN2,LN3,LN4.**Step 5:** Picking three LNs from LN1,LN2,LN3,and LN4 to draw three circles, the cases contain (LN1,LN2,LN3), (LN1,LN2,LN4), (LN1,LN3,LN4) and (LN2,LN3,LN4), four geometric centers of the intersection area can be obtained expressed as $\left(x_{123},y_{123}\right)$, $\left(x_{124},y_{124}\right)$, $\left(x_{134},y_{134}\right)$, and $\left(x_{234},y_{234}\right)$.**Step 6:** Calculate the average value of $\left(x_{123},y_{123}\right)$,$\left(x_{124},y_{124}\right)$, $\left(x_{134},y_{134}\right)$, $\left(x_{234},y_{234}\right)$ as the coordinate of the UN.
$$\left(x,y)=(\frac{x_{123}+x_{124}+x_{134}+x_{234}}{4},\frac{y_{123}+y_{124}+y_{134}+y_{234}}{4}\right)$$

## 6. The Extension of CRFLA

**Figure 6:**
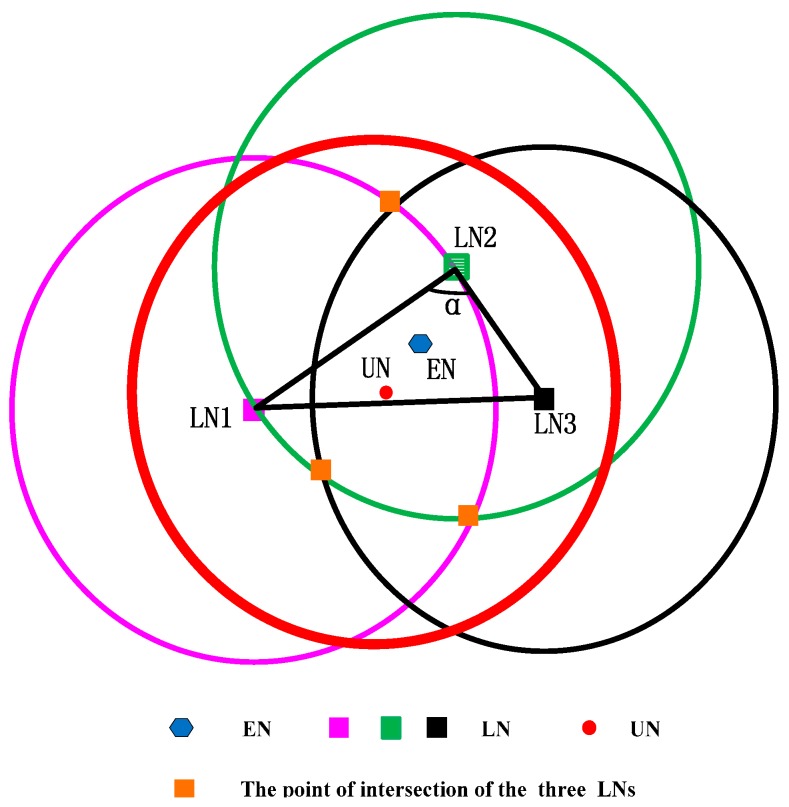
The localization relationship of UN and EN when LN2 is above UN and EN.

Based on CRFLA, we can get an estimation of the unlocalized sensor node’s position. However, the precision is rough. Thus, we try to increase the accuracy of CRFLA by studying the relative relationship between the original coordinates of the unlocalized sensor node, estimation coordinates, and the coordinates of the localized sensor nodes. Considering several of the geometric position relationships of the three coordinates, two cases are shown, as follows: in Figure 6, LN expresses the localized sensor node, and UN expresses the unlocalized sensor node, and EN expresses the estimated coordinate of the unlocalized sensor node. 

Connecting three LN points constitute a triangle. From Figure 6, we can see that EN is far away from UN and close to LN2. Hence, we approximate that EN is in the direction of LN2 away from UN. Figure 6 shows the case that LN2 is above UN and EN.

**Figure 7:**
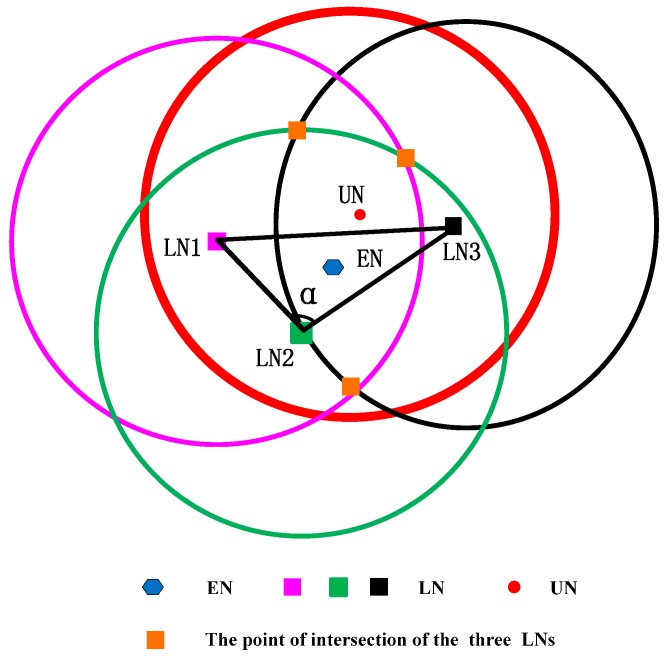
The localization relationship of UN and EN when LN2 is below UN and EN.

The case that LN2 is below EN and UN is shown in Figure 7. Similarly, we can approximate that EN is in the direction of LN2 away from UN. Next, we should try to find out why the point is not LN1 or LN3 but LN2. Through observation, we find the angle α is the largest angle in the triangle. We test some cases and from those tests, it is true that it is a high possibility that EN is in the direction of LN2 away from UN. Hence, LN2 corresponds to the point that the angle of it is the largest angle in the triangle. We transfer the largest angle to the longest opposite edge. It means that the distance between LN1 and LN3 is the largest. We will use the MATLAB simulation to demonstrate it. Next, we give the mathematical model of the extension of CRFLA.

The coordinate of UN is (x,y,z), and the coordinate of LN2 is $\left(x_{2},y_{2},z_{2}\right)$, and the coordinate of EN is $\left(\hat{x},\hat{y},\hat{z}\right)$. If we want $\left(\hat{x},\hat{y},\hat{z}\right)$ is close to (x,y,z), we should adjust the coordinate $\left(\hat{x},\hat{y},\hat{z}\right)$ in the opposite direction of the movement of EN where EN is in the direction of LN2 away from UN. Because the z-coordinate can be obtained from the pressure sensor equipped on the unlocalized sensor node, we just discuss the adjustment of the x-coordinate and y-coordinate. The final coordinate of the unlocalized sensor node can be formulated as follows.

(21) $$\left(x,y)=(\hat{x}\pm a_{1},\hat{y}\pm b_{1}\right)$$

The extension of CRFLA adjusts the estimated coordinates in two steps:

*Step 1*: The adjustment of the x-coordinate:
    *Case I*: if $x_{2}>\hat{x}$, then $x=\hat{x}-a_{1}$;
    *Case II*: if $x_{2}<\hat{x}$, then $x=\hat{x}+a_{1}$;
*Step 2*: The adjustment of the y-coordinate:
    *Case I*: if $y_{2}>\hat{y}$, then $y=\hat{y}-b_{1}$;
    *Case II***:** if $y_{2}<\hat{y}$, then $y=\hat{y}+b_{1}$;

The determination of the variables $a_{1}$ and $b_{1}$ is hard. The value of variables $a_{1}$ and $b_{1}$ will seriously affect the precision of the location. Unfortunately, we still cannot find an excellent method to determine the value of variables $a_{1}$ and $b_{1}$. We first assume a kind of relationship between the variables $a_{1}$, $b_{1}$ and the distance between the side lengths of the triangle drawn by using the localized sensor node. Then we use a significant number of MATLAB simulations to change the parameters to observe the precision changes. In this paper, we use the parameters as follows:

(22) $$a_{1}=\sqrt{\left|x_{2}-\hat{x}\right|}$$

(23) $$b_{1}=\sqrt{\left|y_{2}-\hat{y}\right|}$$

We will show the comparison of the different parameters using a MATLAB simulation.

## 7. TP-TSFLA Procedure

The TP-TSFLA proposed in this paper contains two phases. In Phase I, the mobile beacon is employed as the anchor to realize the time synchronization-free localization of sensor nodes. If the sensor node cannot obtain its coordinates in Phase I, it goes into Phase II and uses CRFLA to locate the unlocalized sensor nodes. Hence, for each phase, there are two steps. The detailed algorithm can be described as follows:

Phase I: Range-based estimation phase

*Step 1*: The sensor node uses the TSFL algorithm to obtain the distance measure d from the mobile beacon to the sensor nodes. In this step, sensor nodes passively listen to the messages of the mobile beacon. Then using those messages received from the mobile beacons, the sensor node can measure the distance from the mobile beacon. If at least three distance measures are obtained, sensor node records those distance measures and goes into Step 2.

*Step 2*: Every sensor node which has obtained at least three distance measures through Step 1 use the PSO algorithm to obtain the estimated position.

Phase II: Range-free evaluation phase

*Step 3*: Sensor nodes which cannot obtain their position through Phase I actively launch the localization request. The localized sensor nodes within the transmission range of the unlocalized sensor node receive the request and respond their coordinates to the sensor node. Then the unlocalized sensor node uses CRFLA to obtain its coordinates.

*Step 4*: Sensor nodes adjust the estimated coordinate to improve the precision of the range-free method, and the final estimated location is taken as the coordinate of the unlocalized sensor node.

**Figure 8:**
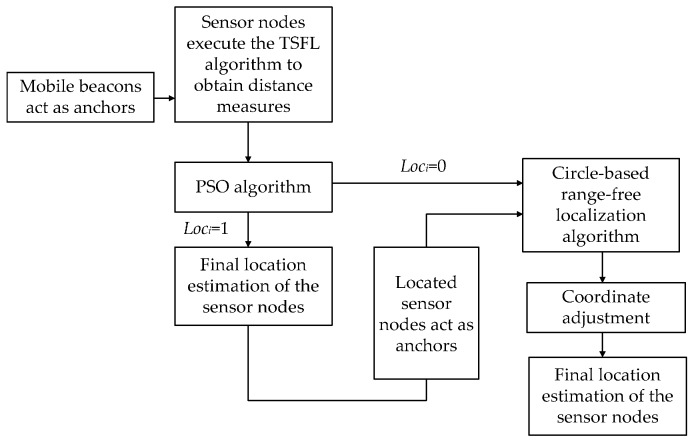
Block diagram of TP-TSFLA.

The block diagram of TP-TSFLA is shown in Figure 8.

Table 2 gives all the mathematical notation and symbols definitions used in Algorithm 2.

The detailed stepwise procedure for TP-TSFLA is shown in the following Algorithm 2.
**Algorithm 2:** TP-TSFLA**Phase I:**
**Step 1: TSFL**1: Each sensor node i initialize data: $z_{3}=depth$, $M_{ii}$ = 0, $Loc_{i}$ = 0, $D_{i}$ = 02: Sensor node receives the beacons and records the beacon id j, $M_{j}$ = $M_{j}$ + 13: if $M_{j}$ ≥ 2, sensor node uses TSFL algorithm to compute the distance measure $d_{i}$, records $D_{i}$ = $D_{i}$ + 14: **end if****Step 2: The PSO algorithm**5: **if**
$D_{i}\geq 3$6: Initialize the parameter of PSO, and produce initial particles jj and velocities, and compute $f_{jj}$, then set Maxgen7: **while** the Maxgen is not achieved **do**8: Update the velocity, particle population, and $f_{jj}$9: Update the population optimal gbest10: **end while**11: $coordinate_{i}$ = gbest, sensor node records $Loc_{i}$ = 112: **end if****Phase II:****Step 3: Circle based range-free localization**13: **if**
$Loc_{i}$ = 014: $coordinate\_initial_{i}$ = **Algorithm I**15: $Loc_{i}$ = 1**Step 4: Sensor nodes adjust the estimated coordinates to improve the precision**16: Compute the parameter $a_{1}=\sqrt{\left|x_{1}-\hat{x}\right|}$, $b_{1}=\sqrt{\left|y_{2}-\hat{y}\right|}$17: Adjust the x-coordinate and y-coordinate: $x=\hat{x}\pm a_{1}$, $y=\hat{y}\pm b_{1}$18: $coordinate_{i}=(x,y,z_{3})$19: **end if**

## 8. Discussion

In this section, we use a MATLAB simulation to evaluate the performance of TP-TSFLA. The simulation environment is 600 m × 600 m × 500 m. In order to sense the data of the whole simulation environment, we use 800 sensor nodes (the transmission range of sensor nodes is tens of meters) in UWSNs. All sensor nodes are regarded as stationary. Considering the size of the simulation environment, balancing the localization ratio and cost, we use 25 mobile beacons in this environment. The following parameters are the same as [12]. The speed of sound is set to 1500 m/s, and the rate of the mobile beacon is 1 m/s. The beacon interval varies from the 30 s to 100 s, and the transmission range varies from 150 m to 250 m.

**Figure 9:**
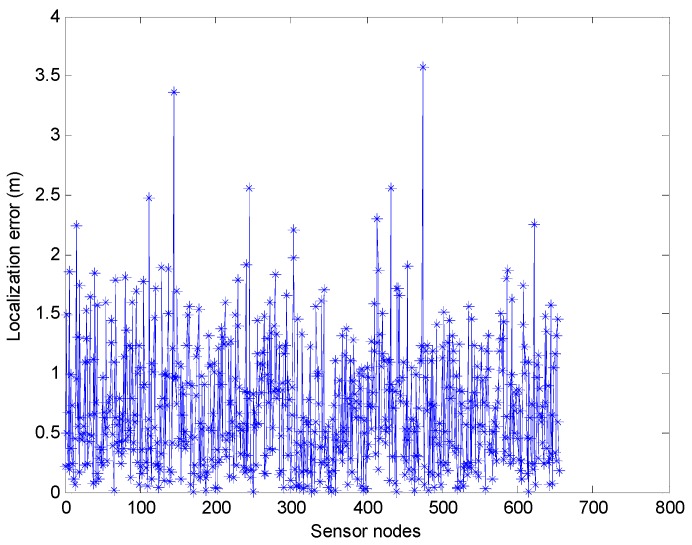
The localization error of the range-based estimation algorithm of using PSO.

In [12], the authors do not show the localization precision of their algorithm. The effect of the underwater environment is not taken into account. Here we consider the factors which may lead to the distance measurement error. Thus, we employ the PSO algorithm to obtain the coordinate of the unknown sensor nodes. The maximum number of iterations $k_{\max}$ is 200, the search space of the particle is the union set of the several circles whose center is the mobile beacon and the radius is the transmission range R. Because the transmission range R is large, thus the search space is relatively large. Thus, particle number has a big impact on average localization error of the algorithm. Here the particle number varies from 100 to 1000. And we design the experiment to compare the localization time and the average localization error at different particle number. The inertia weight can be obtained by using the Equation (16). In our experiment simulation, $\omega_{\max}=0.9,\;\omega_{\min}=0.4$. $c_{1}$ and $c_{2}$ can be calculated by Equations (17) and (18), and $k_{\max}=200$. First, we fix the number of particles at 600, and the localization error of the sensor nodes by using the range-based estimation algorithm is shown in Figure 9. The average positioning error of the range-based estimation algorithm of using PSO by the MATLAB simulation is 0.7123 m. Note that we use 800 sensor nodes in this simulation, but in Figure 9 only about 650 sensor nodes are shown. It is because that some sensor nodes cannot obtain the position information only through Phase I. The localization ratio of the range-based estimation algorithm of using PSO is about 82.13%.

**Figure 10:**
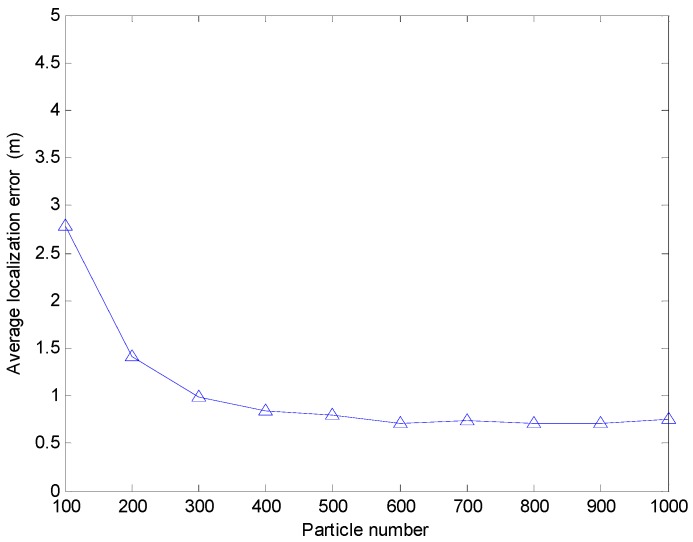
The average localization error with different parameter setting in the PSO algorithm.

**Figure 11:**
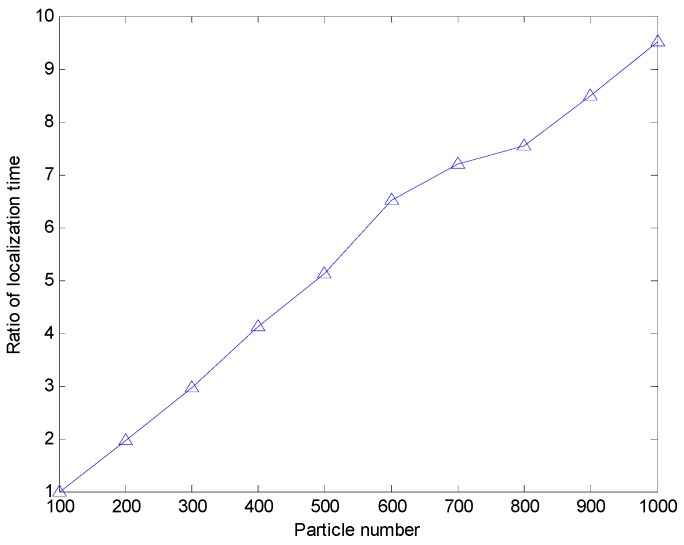
The ratio of localization time with different parameter settings in the PSO algorithm.

Meanwhile, we discuss the effect of the particle number on the localization error when the sensor node uses the range-based estimation algorithm of using PSO to realize localization. Here we make the assumption that the particle number varies from 100 to 1000. Due to the large search space, the right choice of the particle number will decrease the average localization error. Moreover, the more particles mean the longer positioning time. In this simulation, we record the ratio of the localization time $Tr_{i}=\frac{T_{i}}{T_{100}}$. $T_{i}$ is the localization time when particle number is i(i=100,200,300,...,1000). When the particle number varies from 100 to 1000, the average localization error and the ratio of the localization time of the range-based estimation algorithm of using PSO is shown in Figure 10 and Figure 11, respectively. From Figure 10, we can see the average localization error is larger when the particle number is less than 600. While the particle number is more than 600, the increase of particle number does not significantly improve the positioning accuracy. From Figure 11, we can see that the localization time increase greatly with the increase of particle number. Thus, it is not worth increasing the more particles to decrease the localization error when the particle number is more than 600. Even the average error localization will increase with the increase of the particle number when the particle number is more than 600. It may be caused by the PSO algorithm falling into a local optimum.

**Figure 12:**
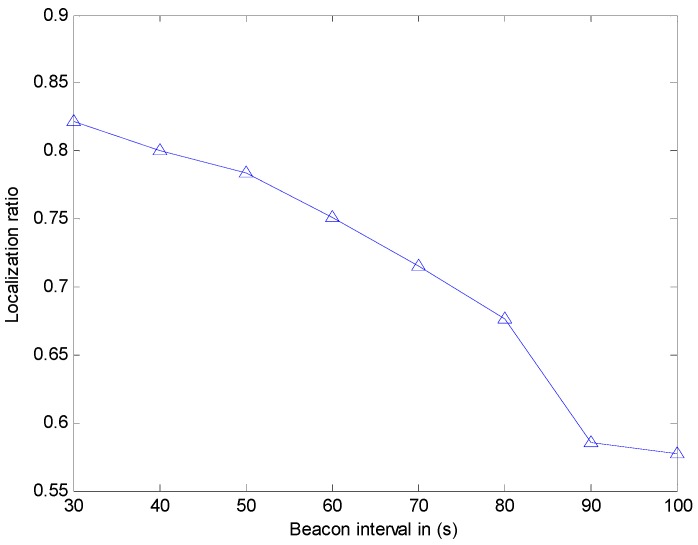
The localization ratio versus beacon interval.

**Figure 13:**
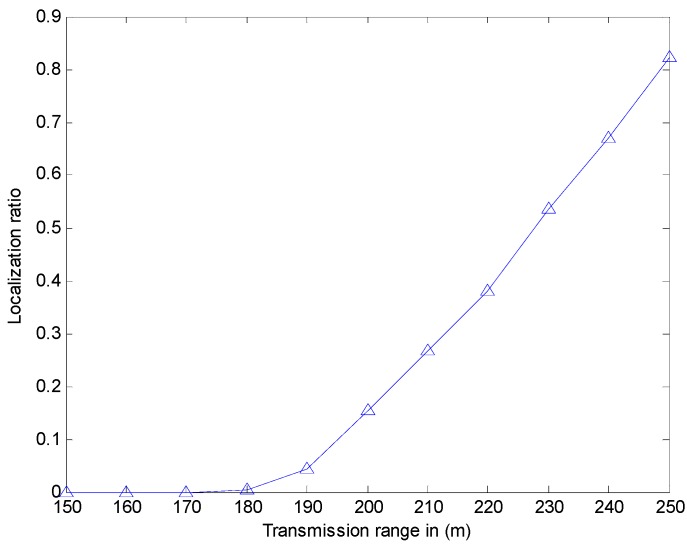
The localization ratio versus transmission range.

We study the effect of the beacon interval and transmission range on the localization ratio (defined as the number of localized sensor nodes). However, in [12], the authors did not put out the simulation environment, just noting that sensor nodes are 250. In [12], the authors show that the localization ratio is about 76% when the beacon interval is 100 s, while the localization ratio is about 90% when the beacon interval is 30 s. However, our simulation experiment (shown in Figure 12) indicates that the localization ratio is about 57.75% when the beacon interval is 100 s, while the localization ratio is 82.13% when the beacon interval is 30 s. In [12], when the transmission range is 150 m, the localization ratio is about 80%, while the transmission range is 250 m, the localization ratio is about 90%. However, our simulation experiment (shown in Figure 13) indicates that the localization ratio is close to 0 when the transmission range is small than 180 m. Hence, the localization ratio is close to 82.13% when the transmission range is 250 m. The reason for it may be that our simulation environment is much larger than that used in [12]. Meanwhile, the results show that when this method is utilized in the larger environment, the localization ratio may be not enough. Thus, we use CRFLA to improve the localization ratio. In the CRFLA, we use the beacon interval as 30 s and the transmission range as 250 m.

**Figure 14:**
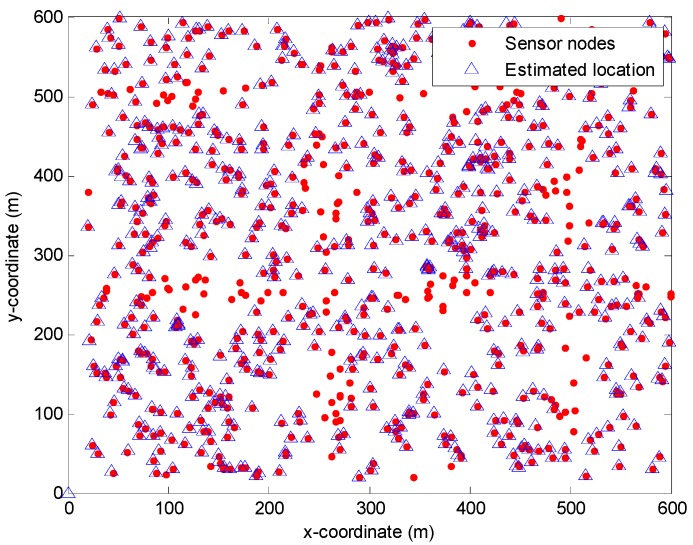
The location of the sensor node projected to the 2D-plane.

CRFLA is based on the assumption that the number of anchor nodes is relatively larger in the localized-to-be area. In TP-TSFLA, only x-coordinate and y-coordinate of the sensor nodes are needed to obtain. Therefore, we project sensor nodes into the 2D-plane and study the 2D-relationship between sensor nodes. In Figure 14, we use the red circle to express the sensor node, and the blue triangle to show the estimation location of the range-based estimation algorithm of using PSO. We can see the red circle which is not surrounded by the blue triangle is the unlocalized sensor nodes. Hence, it is evident that the localized sensor nodes (new anchor nodes) are much more than the unlocalized sensor nodes. They locate in the different directions of the unlocalized sensor nodes. Thus, the prerequisites of CRFLA are established.

**Figure 15:**
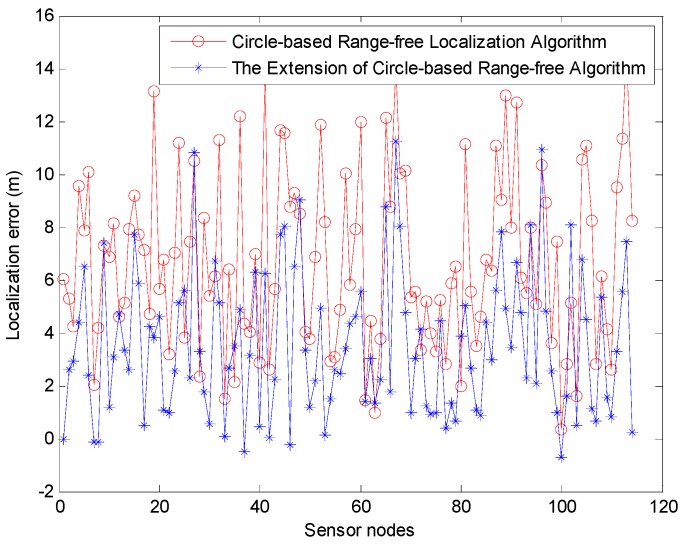
The localization error of CRFLA.

We then use the MATLAB simulation to estimate the positioning error of CRFLA. The results show that the average positioning error is about 6.7996 m. Compared with the range-based localization algorithm (Phase I), the localization error is much larger. This is the shortcoming of the range-free localization algorithms, but the range-free localization is much simpler, and the power consumption is much lower. Besides, the range-free localization does not need some unrealistic assumptions such as precise time synchronization, and fixed speed which may lead to the localization error. An extension of CRFLA is proposed by designing a coordinate adjustment scheme. The comparison of the localization error of the unlocalized sensor node in Phase II is shown in Figure 15. 

From Figure 15, we can see most of the localization error of the extension of CRFLA is lower than CRFLA. The average positioning error using the extension of CRFLA is 3.5348 m. Besides, the extension of CRFLA may increase the localization error. But it is just a small part of it. Hence, to this extent, the coordinate adjustment scheme is useful. Compared with TSFL which those coordinates of unlocalized sensor node are unknown, TP-TSFLA can locate most of them with the average localization error of 3.5348 m and has significantly improved the performance. The localization ratio is 96.38%, while the localization ratio of TSFL is 82.13%.

**Figure 16:**
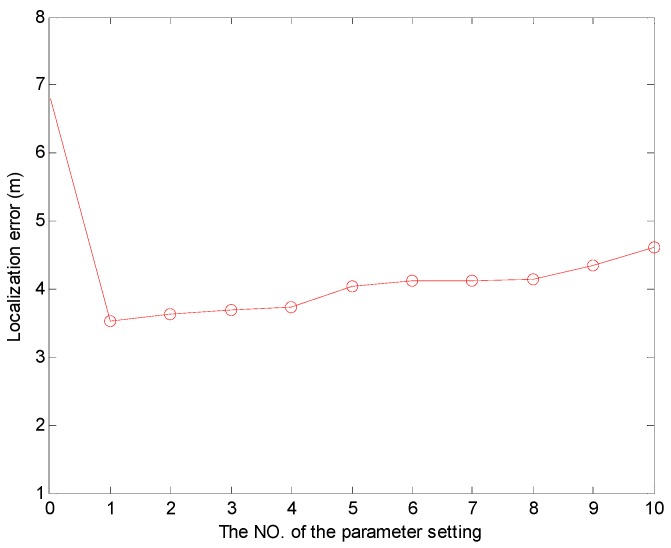
The localization error of different coordinate adjustment scheme parameters $a_{1}$, and $b_{1}$.

Moreover, we survey the effect of the coordinate adjustment parameter settings on the localization error. We discuss some cases, and here we just list ten of them whose localization error is relatively small. We list the parameter settings of $a_{1}$ and $b_{1}$, and the No (#) of the cases, and the different localization errors are shown in Table 3. The coordinates of the UN are (x,y,z), the coordinates of LN1 are $\left(x_{1},y_{1},z_{1}\right)$, the coordinates of LN2 are $\left(x_{2},y_{2},z_{2}\right)$, the coordinates of LN3 are $\left(x_{3},y_{3},z_{3}\right)$, and the coordinates of EN are $\left(\hat{x},\hat{y},\hat{z}\right)$. Note that LN2 corresponds to the point where the angle is the largest angle in the triangle. We use the No (#) of cases as 0 to express CRFLA. Then we discuss some cases of the parameter settings and choose ten of them to show the localization error. We can see the different parameter settings can decrease the localization error at different extent. Compared with the localization error of CRFLA, the localization error of all the cases is reduced and shown in Figure 16.

The MATLAB simulation shows that the average localization error of the range-based estimation algorithm by using PSO is 0.7123 m. The PSO algorithm can efficiently locate the sensor nodes and ensure a relatively high accuracy without time synchronization. In Phase II, we use CRFLA to locate the unlocalized sensor nodes, and the localization ratio achieved is 96.38% while the localization ratio using TSFL is 82.13%. The average localization error of CRFLA is 6.7996 m while using the coordinate adjustment scheme so the average localization error can decrease to 3.5348 m.

## 9. Conclusions

To make better use of underwater resources and realize the application of UWSNs, the localization of sensor nodes for UWSNs is the critical issue. Many scholars have put forward different localization techniques for UWSNs. However, most of them are based on the assumption of accurate synchronization between sensor nodes. In fact, this is tough to achieve. The TP-TSFLA method proposed in this paper contains two phases, namely, a range-based estimation phase and a range-free evaluation phase. First, we use the TSFL algorithm to obtain the distance measurements from the mobile beacons to the sensor nodes. Then the PSO algorithm is employed to estimate the location of the sensor nodes. Moreover, CRFLA locates the unlocalized sensor nodes after Phase I. We use a multi-stage scheme where the localized sensor nodes acted as the new anchor nodes to help realize localization. Besides, a coordinate adjustment scheme is extended to improve the precision of the circle-based range-free algorithm. The simulation results show that TP-TSFLA can achieve a relative localization ratio without time synchronization and the coordinate adjustment scheme can decrease the localization error. However, there are still some issues that demand further study. We design the two conditions based on experience and experiments. therefore, it just can only guarantee with a high probability that the selected anchor nodes are optimal. We will further improve the two conditions. If the coordinate adjustment scheme is designed more reasonable, the localization error will decrease a lot. Hence, we will find the better parameter setting of the coordinate adjustment scheme. The impact of the localization protocols on the routing and clustering protocols is also a direction in the future.

## Figures and Tables

**Table 1:** The comparison of the localization algorithms.

Reference	Computation Algorithm	Anchor Requirement	Range Measurement	Synchronization Requirement	Communication between Nodes
[17]	Centralized	Anchor-based	Range-based	Synchronization	Single stage
[18]	Distributed	Anchor-based	Range-based	Not specified	Single stage
[19]	Hybrid	Anchor-based	Hybrid	Not specified	Single stage
[20]	Distributed	Anchor-free	Range-based	Synchronization	Single stage
[16]	Distributed	Anchor-based	Range-based	Synchronization	Multi-stage
[21]	Centralized	Anchor-based	Range-free	Synchronization-free	Multi-stage
[22]	Centralized	Anchor-based	Range-based	Synchronization	Single stage
[23,24]	Distributed	Anchor-based	Range-free	Synchronization-free	Single stage
[25]	Distributed	Anchor-based	Range-based	Synchronization-free	Multi-stage
[26]	Distributed	Anchor-based	Range-based	Not specified	Single stage
[27]	Distributed	Anchor-based	Range-based	Synchronization	Single stage
[28]	Distributed	Anchor-based	Range-based	Synchronization	Multi-stage
[29]	Distributed	Anchor-based	Range-based	Synchronization	Multi-stage
[30]	Distributed	Anchor-based	Range-based	Synchronization	Multi-stage
[32]	Centralized	Anchor-based	Range-based	Synchronization	Multi-stage
[33]	Distributed	Anchor-based	Range-free	Not specified	Multi-stage
Our	Distributed	Anchor-based	Hybrid	Synchronization-free	Multi-stage

**Table 2:** Notation.

Sign	Meaning
$D_{i}$	Number of mobile beacons to which the distance measurement from the sensor node i is available
$M_{j}$	The number of the message received from the j mobile beacon
$Loc_{i}$	If the node i is localized, the $Loc_{i}=1$, else $Loc_{i}=0$
$z_{3}$	The z-coordinate of the sensor node
$\begin{array}{c} coordinate_{i}\\ \left(x,y,z_{3}\right) \end{array}$	The estimated coordinate of the sensor node i after using the optimized algorithm
$f_{jj}$	The fitness function of the particle jj
Maxgen	Number of iterations
gbest	The population optimal
$\left(x_{2},y_{2},z_{2}\right)$	The coordinate of localized sensor node which corresponds to the point that the angle of it is the largest angle in the triangle

**Table 3:** The parameter setting of the coordinate adjustment scheme and their own localization error.

No (#)	The Parameter Setting of $a_{1}$ and $b_{1}$	Average Localization Error
0	$a_{1}=b_{1}=0$	6.7996 m
1	$a_{1}=\sqrt{\left|x_{2}-\hat{x}\right|}$ $b_{1}=\sqrt{\left|y_{2}-\hat{y}\right|}$	3.5348 m
2	$a_{1}=\sqrt{\left|x_{2}-\hat{x}\right|}+2$ $b_{1}=\sqrt{\left|y_{2}-\hat{y}\right|}+1$	3.6220 m
3	$a_{1}=\left|x_{2}-\hat{x}\right|÷5$ $b_{1}=\left|y_{2}-\hat{y}\right|÷5$	3.6815 m
4	$a_{1}=\left(\sqrt{\left|x_{1}-\hat{x}\right|}+\sqrt{\left|x_{2}-\hat{x}\right|}+\sqrt{\left|x_{3}-\hat{x}\right|}\right)÷3$ $b_{1}=\left(\sqrt{\left|y_{1}-\hat{y}\right|}+\sqrt{\left|y_{2}-\hat{y}\right|}+\sqrt{\left|y_{3}-\hat{y}\right|}\right)÷3$	3.7380 m
5	$a_{1}=b_{1}=\left(\begin{array}{l} \sqrt{{\left(x_{1}-\hat{x}\right)}^{2}+{\left(y_{1}-\hat{y}\right)}^{2}}+\sqrt{{\left(x_{2}-\hat{x}\right)}^{2}+{\left(y_{2}-\hat{y}\right)}^{2}}\\ +\sqrt{{\left(x_{3}-\hat{x}\right)}^{2}+{\left(y_{3}-\hat{y}\right)}^{2}} \end{array}\right)÷25$	4.0327 m
6	$a_{1}=b_{1}=\left(\begin{array}{l} \sqrt{{\left(x_{1}-\hat{x}\right)}^{2}+{\left(y_{1}-\hat{y}\right)}^{2}}+\sqrt{{\left(x_{2}-\hat{x}\right)}^{2}+{\left(y_{2}-\hat{y}\right)}^{2}}\\ +\sqrt{{\left(x_{3}-\hat{x}\right)}^{2}+{\left(y_{3}-\hat{y}\right)}^{2}} \end{array}\right)÷40$	4.1148 m
7	$a_{1}=b_{1}=\left(\begin{array}{l} \sqrt{{\left(x_{1}-x_{2}\right)}^{2}+{\left(y_{1}-y_{2}\right)}^{2}}+\sqrt{{\left(x_{1}-x_{3}\right)}^{2}+{\left(y_{1}-y_{3}\right)}^{2}}\\ +\sqrt{{\left(x_{3}-x_{2}\right)}^{2}+{\left(y_{3}-y_{2}\right)}^{2}} \end{array}\right)÷40$	4.1152 m
8	$a_{1}=b_{1}=\left(\left|x_{2}-\hat{x}\right|+\left|y_{2}-\hat{y}\right|\right)÷15$	4.1489 m
9	$a_{1}=b_{1}=\sqrt{{\left(x_{2}-\hat{x}\right)}^{2}+{\left(y_{2}-\hat{y}\right)}^{2}}÷15$	4.3465 m
10	$a_{1}=\left(\left|x_{1}-x_{2}\right|+\left|x_{1}-x_{3}\right|\right)÷16$ $b1=\left(\left|y_{1}-y_{2}\right|+\left|y_{1}-y_{3}\right|\right)÷16$	4.6065 m
